# Statin use is associated with a lower risk of all-cause death in patients with breast cancer treated with anthracycline containing regimens: a global federated health database analysis

**DOI:** 10.1007/s10238-024-01395-z

**Published:** 2024-06-12

**Authors:** Tommaso Bucci, Ying Gue, Rebecca Dobson, Carlo Palmieri, Pasquale Pignatelli, Gregory Y. H. Lip

**Affiliations:** 1 Liverpool Centre of Cardiovascular Science at University of Liverpool, Liverpool John Moores University and Liverpool Heart and Chest Hospital, Liverpool, UK; 2 Department of Clinical Internal, Anesthesiologic and Cardiovascular Sciences, Sapienza University of Rome, Rome, Italy; 3 Department of Molecular and Clinical Cancer Medicine, Institute of Translational Medicine, University of Liverpool, Liverpool, UK; 4 The Clatterbridge Cancer Centre NHS Foundation Trust, Liverpool, UK; 5 Department of Clinical Medicine, Danish Center for Health Services Research, Aalborg University, Aalborg, Denmark

**Keywords:** Breast cancer, Statins, Cardiovascular events, Mortality, Anthracyclines

## Abstract

Anthracyclines are associated with enhanced oxidative stress responsible for adverse events in patients with breast cancer. However, no study has investigated the potential anti-inflammatory role of statins in counteracting anthracycline toxicity. In this retrospective study utilizing a federated health network (TriNetX), patients with breast cancer (ICD code C50) treated with anthracyclines were categorized into two groups: *statin users* (for at least 6 months); and *statin non-users*. The primary outcome was the 5-year risk of all-cause death. Secondary outcomes were the risk of myocardial infarction, stroke, atrial fibrillation, ventricular arrhythmias, heart failure, and pulmonary embolism. Cox-regression analyses were used to produce hazard ratios (HRs) and 95% confidence intervals (CI) following 1:1 propensity score matching (PSM). We identified 3,701 *statin users* (68.8 ± 10.4 years) and 37,185 *statin non-users* (59.6 ± 12.8 years). After PSM, the 5-year risk of all-cause death was significantly lower in *statin users* (HR 0.82, 95% CI 0.74–0.91) compared to statins non-users. Analyzing the risk for secondary outcomes, only the risk of stroke was significantly increased in *statin users* (HR 1.27, 95% CI 1.01–1.61), while no associations were found for the other cardiovascular events. The risk of all-cause death in *statin users* was the lowest during the first year after the anthracycline’s initiation. No significant difference was found between lipophilic and hydrophilic statins. In patients with breast cancer treated with anthracyclines, statin use is associated with a reduced risk of all-cause death. Prospective studies are needed to investigate the potential beneficial effect of statin initiation in cancer patients without other indications.

**Supplementary Information:**

The online version contains supplementary material available at 10.1007/s10238-024-01395-z.

## Introduction

Anthracyclines have been the mainstay therapy for breast cancer for many decades, reducing recurrence and improving survival in patients with breast cancer [1, 2]. However, adverse cardiovascular effects of anthracyclines, particularly reduction in left ventricular systolic function related to the cumulative dose-dependent effect may be a limiting factor [3].

Statins play an integral role in the management of cardiovascular risk in the regulation of cholesterol levels through inhibition of 3-hydroxy-3-methylglutaryl-coenzyme-A reductase (HMG-CoA-R), predominantly within the hepatocytes [4]. Apart from its benefits in modulating lipids, statins have also shown promise in their anti-cancer effects [5, 6], particularly in breast cancer where the HMG-CoA-R overexpression has previously been shown [7–9]. Although there have been multiple studies showing the beneficial effect statins have on mortality in patients with breast cancer [10–13], there were also studies, particularly outside of Scandinavian populations [14, 15] where the beneficial effects of statins are less prominent.

In a meta-analysis exploring use of statins in patients with breast cancer, Manthravadi et al*.* reported that statin use, particularly lipophilic statins, were associated with an improvement in recurrence-free survival (Hazard Ratio (HR) 0.72; 95% Confidence Interval (CI) 0.59–0.89). However, this was not seen in patients on hydrophilic statins (HR 0.80; 95% CI 0.44–1.46) [16]. Liu et al. also showed similar trends with cancer-specific and all-cause mortality but only less than 4 years of follow-up [17]. In addition to the mortality benefits, statins have also been associated with reduced cardiotoxicity after anthracycline use [18, 19] and therefore may be of benefit in not only reducing mortality but also cardiovascular adverse events.

As there has not been any studies investigating the effects of statins in patients with breast cancer treated with anthracyclines, we aimed to investigate the effects of statins in this population.

## Methods

This was a retrospective observational study conducted within TriNetX, a global federated health research network with access to electronic medical records (EMRs) from participating health care organizations including academic medical centers and community hospitals covering approximately 70 million individuals, mainly in the United States. Within this network, available data include demographics, diagnoses using International Classification of Diseases, Ninth Revision and Tenth Revision, Clinical Modification (ICD-10-CM) codes, and medications. More information can be found online (https://trinetx.com/company‐overview/).

### Cohort

The searches on the TriNetX online research platform were performed on the 01st of June 2023 for individuals aged ≥ 18 years with breast cancer (termed as Malignant neoplasm of breast ICD-10-CM code C50) treated with anthracyclines. To include the highest number of patients possible, the searches were not restricted to a specific period; however, more than 95% of patients considered in this study were entered in the TriNetX platform between 2000 and 2020. At the time of the search, 80 participating health care organizations had data available for patients who met the study inclusion criteria. The baseline index event date was the date of the first reported treatment with anthracyclines; any diagnoses registered before this date were considered as an individual’s baseline characteristics. According to the presence of a statin therapy during the 6 months before the first anthracyclines treatment, the cohort was divided into groups: *statins users* and *statins non-users*.

### Outcomes

The primary outcome was the 5-year risk of all-cause death after the first anthracycline treatment. Secondary outcomes were the risk of acute myocardial infarction, ischemic stroke, atrial fibrillation, ventricular arrhythmias (ventricular fibrillation, ventricular tachycardia), heart failure, and pulmonary embolism. The occurrence of the primary and secondary outcomes was analyzed based on the statin use before the anthracycline therapy. The cardiac complications of interest were identified via ICD-10-CM code (Supplementary Table 1).

To estimate the risk of a *new cardiac* complication, we performed an exploratory analysis excluding patients who experienced a similar outcome before the anthracycline therapy. Additionally, the risk of primary and secondary outcomes in *statin users* and *non-users* was further assessed in different time frames: 1–6 months (*intra treatment risk*), 6 months-1 year (*early post treatment risk*) and 1–5 years (*long-term risk*).

Moreover, to assess the presence of difference in the 5-year risk of primary outcomes based on the type of statin, we analyzed separately patients treated with *lipophilic* (atorvastatin, simvastatin, lovastatin) and *hydrophilic* (rosuvastatin and pravastatin) statins compared to *statins non-users.*

### Statistical analysis

All statistical analyses were performed on the TriNetX online research platform. Baseline characteristics were compared using chi-squared tests for categorical variables and independent-sample t tests for continuous variables. A competitive risk analysis was performed using the Aalen–Johansen plot to calculate the cumulative incidence of all-cause death and cardiovascular events (acute myocardial infarction, ischemic stroke, atrial fibrillation, ventricular arrhythmias, heart failure, and pulmonary embolism) in patients with breast cancer *statin users* and *non-users*.

We performed 1:1 propensity score matching (PSM) to create balanced cohorts. We included the following variables in the PSM: age, sex, ethnicity, arterial hypertension, ischemic heart diseases, atrial fibrillation, heart failure, dyslipidemia, diabetes, obesity, chronic kidney disease, ischemic stroke, and cardiovascular medications (including anticoagulants, antiplatelets, β-blockers, antiarrhythmics, diuretics, antilipemic agents, antianginals, calcium channel blockers, and angiotensin-converting enzyme inhibitors and angiotensin II inhibitors). After PSM, we used Cox proportional hazard models to calculate HRs and 95% CI for the risk of all-cause death and cardiovascular events based on statins treatment. All tests were two-tailed and *p*-values of ≤ 0.05 were taken to indicate statistical significance. All analyses were performed in the TriNetX platform which uses R’s survival package v3.2-3.

### Falsification endpoints

To quantify the significance of unmeasured bias and confounding, we applied two pre-specified falsification endpoints (endpoints which are not expected to be altered by the interventions; hence, any significant differences are probably due to bias or confounding) (20). These were chosen as pneumonia, unspecified organism (ICD-10-CM J18) and slipping, tripping, stumbling and falls (ICD-10-CM W00-W19).

### Data availability statement and ethical approval

TriNetx is a research network compliant with the Health Insurance Portability and Accountability Act and the US federal law which protects the privacy and security of healthcare data, including de-identified data as per the de-identification standard of the HIPAA Privacy Rule (https://trinetx.com/real-world-resources/publications/). The TriNetX research network is utilized for several scientific purposes and to gain access to the data, a sharing agreement is required. As a federated research network, studies using the TriNetX health research network do not need ethical approval as no patient identifiable identification is received. Further information about the data extraction from TriNetX is reported in the supplementary material.

## Results

**Fig. 1:**
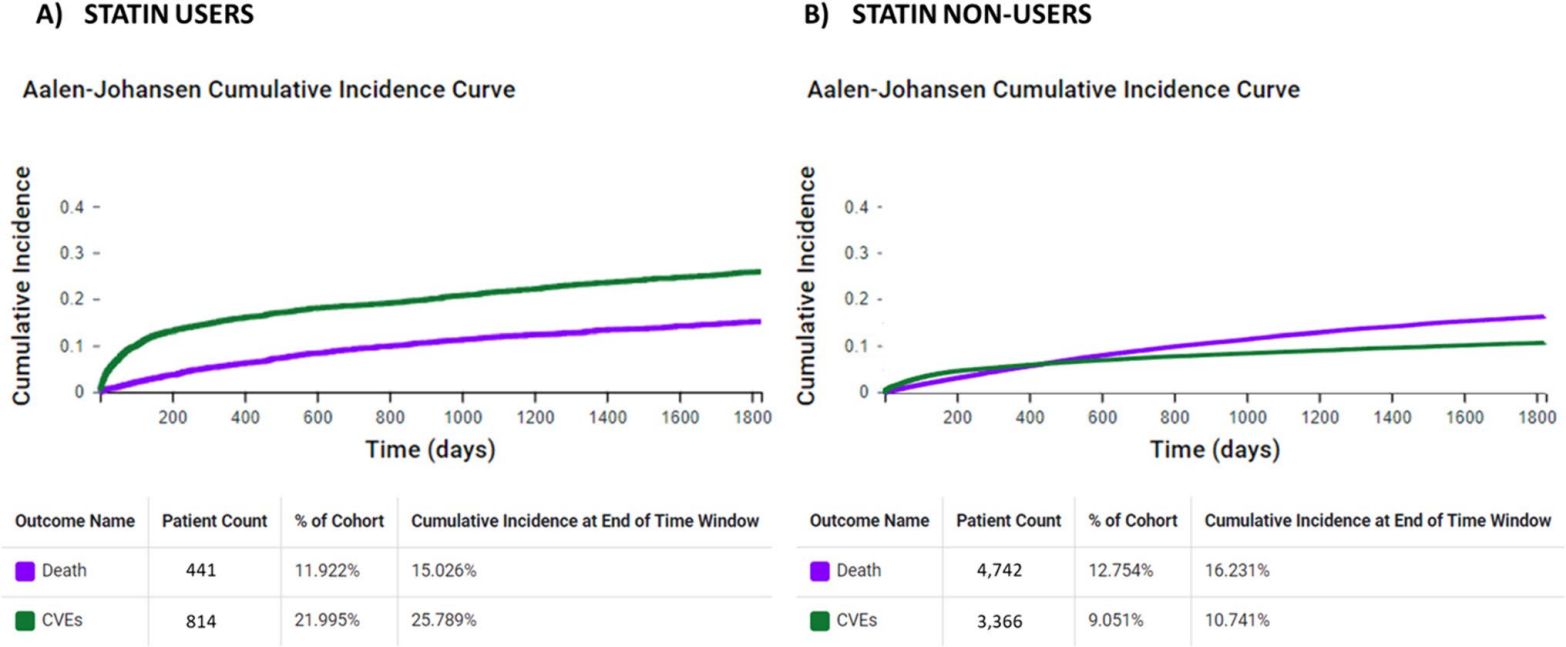
5-year cumulative incidence of all-cause death and cardiovascular events in patients with breast cancer *statin users* (Panel A) and *non-users* (Panel B). *CVEs* Cardiovascular events

The initial cohort consisted of 3,701 patients with breast cancer *statin users* (mean age 59.6 ± 12.8 years, 97.8% females) and 37,185 patients with breast cancer *statin non-users* (mean age 68.8 ± 10.4 years, 98.8% females). Before PSM, *statin users* were older, and with a higher prevalence of atrial fibrillation, heart failure, dyslipidemia, diabetes, obesity, chronic kidney disease, ischemic heart disease and ischemic stroke than *statin non-users* (Table 1). The 5-year cumulative incidences of all-cause death and cardiovascular events in *statin users* were 15.0% and 25.8%, respectively; and in *statin non-users*, 16.2% and 10.7%, respectively. Analyzing the Aalen–Johansen plot, the presence of a crossing point between the incidence projection of all-cause death and cardiovascular events showed the presence of a significant competitive risk between these two outcomes in *statin non-users*, while no significative competitive risk was found among statin users (Fig. 1).

**Table 1:** Baseline characteristics of patients with breast cancer *statin users* and *statin non-users*, before and after propensity score matching

	Before propensity score match			After propensity score match		
	Statin users *n* = 3701	Statin non-users *n* = 37,185	ASD	Statin users *n* = 3315	Statin non-users *n* = 3315	ASD
Age, years (± SD)	68.8 ± 10.4	59.6 ± 12.8	0.790	68.3 ± 10.3	68.7 ± 10.9	0.031
Female, *n* (%)	3453 (97.8)	34,441 (98.8)	0.074	3247 (97.9)	3243 (97.8)	0.008
White, *n* (%)	2361 (66.9)	19,532 (56.0)	0.225	2218 (66.9)	2315 (69.8)	0.063
Black or African American, *n* (%)	596 (16.9)	4025 (11.5)	0.153	553 (16.7)	536 (16.2)	0.014
Arterial hypertension, *n* (%)	2278 (64.6)	7479 (21.5)	0.967	2071 (62.5)	20.83 (62.8)	0.007
Atrial fibrillation, *n* (%)	183 (5.2)	443 (1.3)	0.223	161 (4.9)	147 (4.4)	0.020
Diabetes mellitus, *n* (%)	1202 (34.1)	2429 (7.0)	0.712	1048 (31.6)	1016 (30.6)	0.021
Chronic kidney disease, *n* (%)	301 (8.5)	558 (1.6)	0.320	252 (7.6)	233 (7.0)	0.022
Obesity, *n* (%)	1080 (30.6)	4141 (11.9)	0.470	977 (29.5)	998 (30.1)	0.014
Dyslipidemia, *n* (%)	2540 (72.0)	4729 (13.6)	1.463	2326 (70.2)	2391 (72.1)	0.043
Ischemic heart disease, *n* (%)	587 (16.6)	995 (2.9)	0.478	486 (14.7)	434 (13.1)	0.045
Heart failure, *n* (%)	246 (7.0)	546 (1.6)	0.270	202 (6.1)	188 (5.7)	0.018
Ischemic stroke, *n* (%)	137 (3.9)	170 (0.5)	0.234	112 (3.4)	89 (2.7)	0.040
Beta-blockers, *n *(%)	1627 (46.1)	5432 (15.6)	0.700	1453 (43.8)	1432 (43.2)	0.013
Diuretics, *n* (%)	1657 (47.0)	4727 (13.6)	0.780	1476 (44.5)	1454 (43.9)	0.013
Antiarrhythmics, *n* (%)	2,526 (71.6)	17,189 (49.3)	0.468	2346 (70.8)	2416 (72.9)	0.047
Calcium channel blockers, *n* (%)	1064 (30.2)	2477 (7.1)	0.620	920 (27.8)	878 (26.5)	0.029
ACE inhibitors, *n* (%)	1273 (36.1)	3150 (9.0)	0.684	1121 (33.8)	1064 (32.1)	0.037
Angiotensin II inhibitors, *n* (%)	846 (24.0)	1799 (5.2)	0.553	735 (22.2)	702 (21.2)	0.024
Anticoagulant, *n* (%)	2222 (63.0)	15,325 (44.0)	0.388	2071 (62.5)	2133 (64.3)	0.039
Antiplatelet, *n* (%)	1561 (44.2)	3709 (10.6)	0.813	1365 (41.2)	1280 (38.6)	0.052

*ACE* angiotensin-converting enzyme, *HR* hazard ratio, *95%CI* confidence interval, *SD* standard deviation, *ASD* absolute standardized mean difference

### 5-year risk of all-cause death and cardiovascular events

**Fig. 2:**
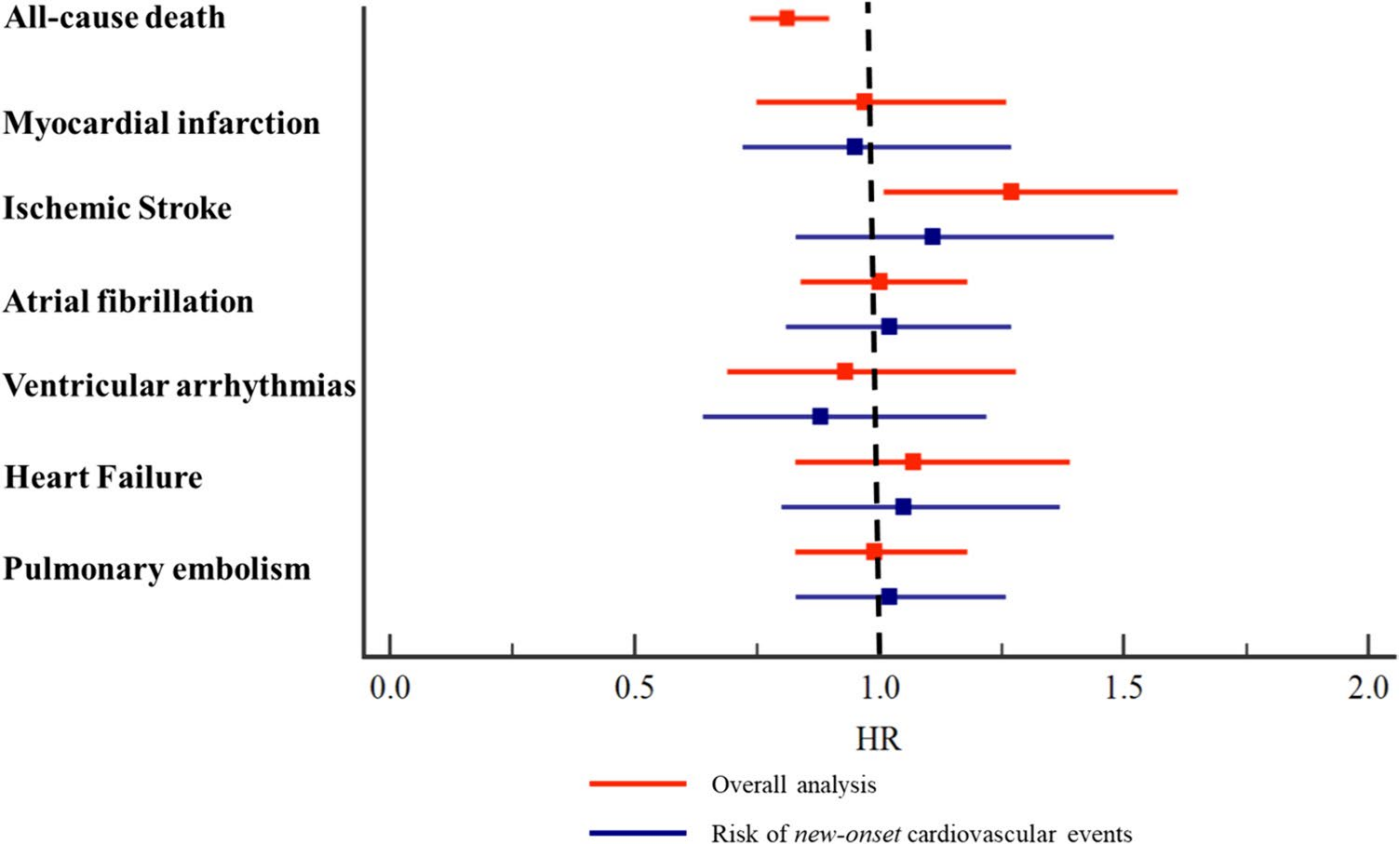
5-year risk of all-cause death and cardiovascular events in patients with breast cancer treated with anthracyclines *statins users* compared to *statin non-users*

After PSM, were identified 3,315 patients for each group and no significative difference was found between the two groups for all the variables considered (Table 1). The primary outcome occurred in 636 (19.2%) patients with breast cancer *statin users* and 744 (22.4%) patients with breast cancer *statin non-users* (HR 0.82, 95%CI 0.74–0.91). For the secondary outcomes, *statin users* showed a higher risk of ischemic stroke (HR 1.27, 95%CI 1.01–1.61) but no significant difference was found for myocardial infarction (HR 0.97, 95%CI 0.75–1.26), atrial fibrillation (HR 1.00, 95%CI 0.84–1.18), ventricular arrhythmias (HR 0.93, 95%CI 0.69–1.28), heart failure (HR 1.07, 95%CI 0.83–1.39), and pulmonary embolism (HR 0.99, 95%CI 0.83–1.18) compared to *statin non-users* (Table 2, Fig. 2).

**Table 2:** 5-year risk of all-cause death and cardiovascular events in patients with breast cancer treated with anthracyclines *statins users* compared to *statin non-users*

	Main analysis after propensity PSM			Sensitivity analysis excluding patients who experienced a CVEs before the anthracyclines treatment				
	Statins users *n* = 3,315	Statins non-users *n* = 3,315		Statin users		Statin non-users		
	Number of events (%)	Number of events (%)	HR (95%CI)	Number of patients	Number of events (%)	Number of patients	Number of events (%)	HR* (95%CI)
All-cause death	636 (19.2)	744 (22.4)	0.82 (0.74–0.91)	–	–	–	–	–
Myocardial infarction	116 (3.5)	116 (3.5)	0.97 (0.75–1.26)	3,227	93 (2.9)	3,260	96 (2.9)	0.95 (0.72–1.27)
Stroke	161 (4.9)	124 (3.7)	1.27 (1.01–1.61)	3,202	98 (3.1)	3,225	86 (2.7)	1.11 (0.83–1.48)
Atrial fibrillation	267 (8.1)	263 (7.9)	1.00 (0.84–1.18)	3,154	156 (4.9)	3,165	149 (4.7)	1.02 (0.81–1.27)
Severe ventricular arrhythmias	78 (2.4)	81 (2.4)	0.93 (0.69–1.28)	3,285	70 (2.1)	3,283	77 (2.3)	0.88 (0.64–1.22)
Heart failure	125 (3.8)	113 (3.4)	1.07 (0.83–1.39)	3,275	112 (3.4)	3,288	104 (3.2)	1.05 (0.80–1.37)
Pulmonary embolism	254 (7.7)	252 (7.6)	0.99 (0.83–1.18)	3,201	188 (5.9)	3,179	178 (5.6)	1.02 (0.83–1.26)

*PSM* propensity score matching, *HR* hazard ratio, *CI* confidence interval, *CVEs* cardiovascular events

### 5-year risk of new-onset cardiovascular events

To estimate the risk of new-onset cardiovascular complications, we performed an exploratory analysis excluding patients who experienced the outcomes of interest before the anthracycline therapy. There were no significant differences in the risks of ischemic stroke (HR 1.11, 95%CI 0.83–1.48), myocardial infarction (HR 0.95, 95%CI 0.72–1.27), atrial fibrillation (HR 1.02, 95%CI 0.81–1.27), ventricular arrhythmias (HR 0.88, 95%CI 0.64–1.22), heart failure (HR 1.05, 95%CI 0.80–1.37), and pulmonary embolism (HR 1.02, 95%CI 0.83–1.26) identified in patients with breast cancer *statin users* compared to those *statin non-users* (Table 2, Fig. 2).

### Sensitivity analysis

Analyzing the different time windows, we found that the risk of primary outcome was significantly reduced during the *intra treatment period* (HR 0.71, 95%CI 0.57–0.89) and in the *early post treatment period* (HR 0.66, 95%CI 0.52–0.83), but was non-significant during *long-term post treatment* (HR 0.95, 95%CI 0.83–1.10) (Table 3). For the secondary outcomes, no significant increased risk was found in *statin users* during the *intra treatment period*, while during the *early post treatment period* there was an increased risk of ischemic stroke (HR 1.57, 95%CI 1.03–2.39) which became non-significant during the *long-term post treatment period* (Table 3). No significant association was found between *statin users* and the other secondary outcomes during the time frames considered (Table 3).

**Table 3:** Risk of primary and secondary outcomes in patients with breast cancer treated with anthracyclines and statins in different time frames

	Intra treatment period (1–6 months)			Early post treatment period (6 months-1 year)			Long-term post treatment group (1–5 years)		
	Statin users	Statin non-users		Statin users	Statin non-users		Statin users	Statin non-users	
	Events *n* (%)	Events *n* (%)	HR 95%CI	Events *n* (%)	Events *n* (%)	HR 95%CI	Events *n* (%)	Events *n* (%)	HR 95%CI
All-cause death	138 (4.2)	193 (5.8)	0.71 (0.57–0.89)	111 (3.3)	165 (5.0)	0.66 (0.52–0.83)	387 (11.7)	386 (11.6)	0.95 (0.83–1.10)
Myocardial infarction	47 (1.4)	47 (1.4)	1.00 (0.67–1.50)	26 (0.8)	21 (0.6)	1.21 (0.68–2.15)	67 (0.2)	66 (0.2)	0.97 (0.69–1.36)
Stroke	62 (1.9)	47 (1.4)	1.32 (0.91–1.94)	56 (1.7)	35 (1.1)	1.57 (1.03–2.39)	116 (3.5)	93 (2.8)	1.20 (0.91–1.57)
Atrial fibrillation	146 (4.4)	141 (4.3)	1.04 (0.82–1.31)	106 (3.2)	98 (3.0)	1.06 (0.81–1.39)	174 (5.2)	168 (5.1)	0.99 (0.80–1.22)
Severe ventricular arrhythmias	29 (0.9)	31 (0.9)	0.93 (0.56–1.55)	18 (0.5)	14 (0.4)	1.26 (0.63–2.53)	46 (1.4)	46 (1.4)	0.95 (0.63–1.43)
Heart failure	40 (1.2)	38 (1.1)	1.05 (0.68–1.64)	30 (0.9)	18 (0.5)	1.63 (0.91–2.93)	77 (2.3)	75 (2.3)	0.98 (0.71–1.35)
Pulmonary embolism	141 (4.3)	152 (4.6)	0.93 (0.74–1.70)	101 (3.0)	99 (3.0)	1.00 (0.76–1.32)	152 (4.6)	127 (3.8)	1.15 (0.91–1.45)

*HR* hazard ratio, *CI* confidence interval

### Lipophilic and hydrophilic statins

After PSM, we identified 2,676 *lipophilic statin users* (68.7 ± 10.3 years, 98% females) and 915 *hydrophilic statin users* (mean age 68.1 ± 10.1 years, 97.8% females) (Supplementary Tables 2 and 3). Compared to *statin non-users*, patients treated with *lipophilic* and *hydrophilic statins* showed a similar risk of all-cause death, which was statistically significant only in the lipophilic group (HR 0.86, 95%CI 0.76–0.96 and HR 0.87, 95%CI 0.71–1.07, respectively) (Table 4). When patients treated with *lipophilic statins* were directly compared to those treated with *hydrophilic statins,* no significant difference was found for the risk of all-cause death (HR 0.98, 95%CI 0.80–1.20).

**Table 4:** 5-year risk of all-cause death in patients with breast cancer treated with anthracyclines based on type of statin

	Number of patients	Number of events (%)	HR (95%CI)
Hydrophilic statins	915	179 (19.6)	0.87 (0.71–1.07)
Statins non-users	915	205 (22.4)	Ref
Lipophilic statins	2,676	523 (19.5)	0.86 (0.76–0.96)
Statins non-users	2,676	581 (21.7)	Ref
Lipophilic statins	914	187 (20.5)	0.98 (0.80–1.20)
Hydrophilic statins	914	180 (19.7)	Ref

*HR* hazard ratio, *CI* confidence interval, *Ref* reference group

### Falsification analysis

After PSM, no statistically significant difference was found for the risk of falsification endpoints between the 2 groups. In particular, pneumonia occurred in 461 (13.9%) *statin users* and 444 (13.4%) *statin non-users* (HR 1.01, 95%CI 0.88–1.15); whereas, slipping, tripping, stumbling and falls occurred in 8.16% *statin users* and 7.44% *statin non-users* (HR 1.06, 95%CI 0.89–1.26).

## Discussion

In this study, our principal findings are as follows: (i) Statin use is associated with an 18% lower all-cause death in patients with breast cancer treated with anthracyclines; (ii) the lower risk of all-cause death in *statin users*, apparently is not associated with a reduced risk of cardiovascular events, (iii) the beneficial effect on mortality of statins in patients with breast cancer treated with anthracyclines is independent of the type of statin (i.e., hydrophilic or lipophilic).

The reduced all-cause mortality in patients with breast cancer *statin users* was particularly evident within the first year of treatment with anthracycline and loses its effect beyond a year of treatment. This was consistent with previous studies exploring the use of statins in breast cancers showing a reduction of 17–54% [10–13]. Since we cannot predict the occurrence of breast cancer, being able to use statins post-diagnosis is particularly important to further improve the prognosis of patients with breast cancer. Scott et al*.* recently found that use of statin post-diagnosis of breast cancer was also associated with a reduction of 26% in risk of breast cancer mortality [20], similar to the paper by Borgquist et al. showing 17% reduction [10]. This provides further support in not only continuing the use of statins but to consider initiation of statins within this patient population. The more evident reduction of the mortality risk just during the *intra treatment* and *early post treatment* period, supports the hypothesis by which the statin treatment effectively counteracts the pro-oxidant side effects associated with the anthracycline treatment. Indeed, the high production of reactive oxygen species is not only associated with cardiotoxicity but also with hepatic failure, tumor lysis syndrome, secondary malignancies (e.g., acute myelogenous leukemia and myelodysplastic syndrome), or myelosuppression that can predispose to infections, sepsis, and death [21, 22]. However, identifying patients who do not satisfy current guidelines for treatment with statins but may benefit from it as part of the treatment for their breast cancer requires further prospective studies and randomized clinical trials. Indeed, although the absence of statistically significant association with the falsification endpoints suggests the absence of concerns about post hoc data mining [23], only a randomized study specifically designed to investigate the possible effect of statin in reducing the risk of death in patients with breast cancer can provide a definitive answer to these open questions.

Although statins have previously been shown to reduce the risk of cardiotoxicity associated with anthracyclines [18, 24, 25], this was not shown in our analysis. Instead, we found an increased risk of stroke and no significant difference in the risk of other cardiovascular events in *statin users* compared to *statin non-users*. The explanations for this finding could lie in several reasons, as follows: (i) in patients with cancer there is a competitive risk between death and cardiovascular events. As shown by the Aalen–Johansen plot, the higher cumulative incidence of death reported in *statin non-users* avoid the development of cardiovascular events during follow-up in this group and could lead to an apparent increased risk of stroke in statin users; (ii) this is a retrospective study in which the statin prescription was done based on the clinical characteristics of patients and not by randomization. Compared to *statin non-users*, *statin users* were at higher risk of cardiovascular events per se for the baseline higher cardiovascular and atherosclerotic burden. These differences could have been only partially mitigated by the PSM because it is based on the balancing of the disease prevalence, whereas no adjustment was possible for disease severity. The exploratory analysis excluding patients with previous cardiovascular events removes the difference between the 2 groups and seems to confirm this hypothesis; and iii) Statins are a well-known class of anti-inflammatory drugs with several pleiotropic effects and a direct antineoplastic action responsible for the lower risk of death in *statin users* cannot be excluded [26]. Moreover, most of the studies that have previously investigated the beneficial effect of statins in preventing the cardiotoxicity of anthracyclines were based on the assessment of the left ventricular ejection fraction by echocardiography, magnetic resonance, or nuclear imaging; all methods that can detect subclinical differences [25, 27–31]. Whereas the few studies that have evaluated the incidence risk of cardiovascular events in breast cancer, reported data just on the risk of new-onset heart failure and were based on selected populations with a significative lower prevalence of cardiovascular risk factors compared to our unselected population [32, 33].

Lastly, the use of lipophilic statins showed a significant reduction in all-cause death when compared to non-statins users; whereas, hydrophilic statins showed a similar but not significant trend in the same comparison. This could be explained by the difference in the group size (*n* = 2,676 vs. 915) where hydrophilic statin users were under-represented leading to a loss of statistical power. Previous pre-clinical studies have demonstrated that statin drug lipophilicity is related with the growth suppressive activity against tumor cells [34, 35]. This has been clinically confirmed also by two different meta-analysis that found a more pronounced beneficial effect in patients treated with lipophilic compared to hydrophilic statins [16, 17]. In our study, the similar magnitude of the risk reduction associated with each type of statin, as well as the absence of significant difference when directly compared, makes it difficult to recommend one over the other.

### Limitations

There are several important limitations to consider for this study. This study was observational and retrospective and despite successful matching we cannot rule out residual bias or indication bias related to unmeasured confounders. Outcomes in this study were based on ICD-10 codes and as such are unadjudicated and may be prone to misclassification or under-reporting. No data are available about the specific cause of death making it impossible to understand the weight of statins in reducing the cancer-related mortality. Given the limited statistical functions of the TriNetX online platform, no interaction analyses were performed to investigate if the magnitude of the risk reduction for all-cause death was statistically significant among the different subgroups. In our study no analysis was done differentiating the risk of adverse events based on statins dosage, lipid lowering intensity and cholesterol levels, and we cannot assess if the statin therapy was taken for all the follow-up period because selecting patients on statin treatment after the index event would have introduced an immortal bias. Moreover, the small number of events do not allow us to assess separately the risk of heart failure with preserved or reduced ejection fraction. No adjustment was done based on the tumor stage, neoplastic receptor profile, as well as for the concomitant use of other antineoplastic therapies that can have contributed to increase the cardiovascular risk. Finally, we just analyzed the risk of cardiovascular events, and no data are available about left ventricular strain or ejection fraction that represents the gold standard to assess the cardiotoxicity associated with anthracycline treatment.

## Conclusion

Statin use was associated with a reduced risk of all-cause death in breast cancer patients treated with anthracycline, supporting the continued use of statins throughout the treatment of breast cancer. Consideration of statin initiation post-diagnosis without other indications would require further studies to identify patients who may benefit from this treatment.

## Supplementary Information

Below is the link to the electronic supplementary material.

Supplementary file1 (DOCX 44 KB)

## Data Availability

The data underlying this article will be shared on reasonable request to the corresponding author.

## Abbreviations

EMRs: Electronic medical records
CI: Confidence interval
CVEs: Cardiovascular events
HMG-CoA-R: 3-Hydroxy-3-methylglutaryl-coenzyme-A reductase
HR: Hazard Ratio
ICD-10-CM: International classification of diseases, tenth revision, clinical modification
PSM: Propensity score matching
