# Supplementary material for: Statin use is associated with a lower risk of all-cause death in patients with breast cancer treated with anthracycline containing regimens: a global federated health database analysis
Source: Clin Exp Med. 2024 Jun 12;24(1):124. doi: 10.1007/s10238-024-01395-z (PMC11168976; doi:10.1007/s10238-024-01395-z)
Supplement: Supplementary file 1 — Supplementary file1 (DOCX 44 KB) [file 10238_2024_1395_MOESM1_ESM.docx]

**Statin use is associated with a lower risk of all-cause death in patients with breast cancer treated with anthracyclines. A global federated health database analysis.**

Tommaso Bucci, Ying Gue, Rebecca Dobson, Carlo Palmieri, Pasquale Pignatelli, Gregory Y. H. Lip

Supplementary data

The TriNetX data are collected from member healthcare organizations (HCO) and originates from their primary electronic health records (EHR) system. A typical HCO is a large academic health center with data coming from majority of its affiliates. A single HCO frequently has more than one facility, including main and satellite hospitals. The data are stored on the TriNetX database via a physical server at the institution’s data centre or a virtual hosted appliance. The TriNetX platform comprises of a series of these appliances connected into a federated network. This network can broadcast queries to each appliance. Results are subsequently collected and aggregated. Once the data are sent to the network, it is mapped to a standard and controlled set of clinical terminologies and undergoes a data quality assessment including ‘data cleaning’ that rejects records which do not meet the TriNetX quality standards. The TriNetX database performs internal and extensive data quality assessment with every refresh based on conformance, completeness, and plausibility (http://doi.org/10.13063/2327-9214.1244). HIPAA (Health Insurance Portability and Accountability Act) compliance of the clinical patient data is achieved using deidentification. Available data types within the network include demographics, diagnoses (represented by ICD-10-CM codes), procedures (coded in ICD-10-PCS or CPT), and measurements (coded to LOINC). While extensive information is provided about patients’ diagnoses and procedures, other variables (such as socioeconomic and lifetime factors are not comprehensively represented). The advantage of electronic health record data over insurance claim data is that both insured and uninsured patients are included. An advantage of electronic health record data over survey data is that the former represents the diagnostic rates in the population presenting to healthcare facilities. This provides an accurate account of the burden of specific diagnoses on healthcare systems. One primary limitation of relying on diagnoses is that they do not account for undiagnosed patients who might have a condition but have not yet received medical support. Another general limitation of electronic health record data is that a patient may be seen in different HCO for different components of their care. If one healthcare organization is not part of the federated network, then part of their medical records may not be available. Using a network of healthcare organizations, rather than a single site, limits this possibility but does not fully remove it. Propensity Score Matched Analyses Using logistic regression [Logistic Regression of the scikit-learn package in Python (version 3.7)], TriNetX performs a 1:1 greedy nearest neighbor matching model, with a caliper of 0.1 pooled standard deviations. To eliminate bias resulting from nearest neighbor algorithms, the orders of rows are randomized. Any baseline characteristic with a standardized mean difference between cohorts lower than 0.1 is deemed well matched (<https://www.tandfonline.com/doi/full/10.1080/00273171.2011.568786>).

Supplementary Table 1. ICD-10-CM codes for primary and secondary outcomes.

| **Early cardiovascular complications** | **ICD-10-CM-codes** |
| --- | --- |
| All-cause death | - Deceased (variable codified by TriNetX). |
| Myocardial infarction | - I21 Acute myocardial infarction |
| Ischemic stroke | - I63 Cerebral infarction |
| Atrial fibrillation | - I48 Atrial fibrillation and flutter |
| Ventricular arrhythmias | - I49.0 Ventricular fibrillation and flutter   and/or   - I47.2 Ventricular tachycardia |
| Acute Heart failure | - I50.21 Acute Systolic heart failure - I50.23 Acute on chronic systolic heart failure - I50.31 Acute diastolic heart failure - I50.33 Acute on chronic diastolic heart failure |
| Pulmonary embolism | - I26 Pulmonary embolism |

Supplementary Table 2. Baseline characteristics of breast cancer patients *hydrophilic statin users* (cohort 1) and *statin non-users* (cohort 2) before and after propensity score matching.

| **Cohort 1 (N = 943) and cohort 2 (N = 37,553) characteristics before propensity score matching** | | | | | | | | | |
| --- | --- | --- | --- | --- | --- | --- | --- | --- | --- |
|  | **Demographics** | | | | | | | | |
|  |  | Cohort | |  | Mean ± SD | Patients | % of Cohort | P-Value | Std diff. |
|  |  | 1 2 | Age | Age | 68.2 +/- 10.1 59.6 +/- 12.8 | 924 35,216 | 100% 100% | <0.001 | 0.748 |
|  |  | 1 2 | 2106-3 | White |  | 617 19,748 | 66.8% 56.1% | <0.001 | 0.221 |
|  |  | 1 2 | F | Female |  | 904 34,766 | 97.8% 98.7% | 0.019 | 0.068 |
|  |  | 1 2 | 2054-5 | Black or African American |  | 173 4,065 | 18.7% 11.5% | <0.001 | 0.201 |
|  |  | 1 2 | 2028-9 | Asian |  | 18 991 | 1.9% 2.8% | 0.115 | 0.057 |
|  | **Diagnosis** | | | | | | | | |
|  |  | Cohort | |  | Mean ± SD | Patients | % of Cohort | P-Value | Std diff. |
|  |  | 1 2 | I10-I16 | Hypertensive diseases |  | 605 7,589 | 65.5% 21.5% | <0.001 | 0.988 |
|  |  | 1 2 | I20-I25 | Ischemic heart diseases |  | 169 1,001 | 18.3% 2.8% | <0.001 | 0.519 |
|  |  | 1 2 | I48 | Atrial fibrillation and flutter |  | 59 450 | 6.4% 1.3% | <0.001 | 0.268 |
|  |  | 1 2 | I50 | Heart failure |  | 74 548 | 8.0% 1.6% | <0.001 | 0.306 |
|  |  | 1 2 | E78 | Dyslipidemia |  | 697 4,818 | 75.4% 13.7% | <0.001 | 1.585 |
|  |  | 1 2 | E08-E13 | Diabetes mellitus |  | 320 2,459 | 34.6% 7.0% | <0.001 | 0.724 |
|  |  | 1 2 | E66 | Obesity |  | 311 4,203 | 33.7% 11.9% | <0.001 | 0.536 |
|  |  | 1 2 | N18 | Chronic kidney disease |  | 90 573 | 9.7% 1.6% | <0.001 | 0.356 |
|  |  | 1 2 | I63 | Cerebral infarction |  | 32 176 | 3.5% 0.5% | <0.001 | 0.214 |
|  | **Medication** | | | | | | | | |
|  |  | Cohort | |  | Mean ± SD | Patients | % of Cohort | P-Value | Std diff. |
|  |  | 1 2 | CV300 | ANTIARRHYTHMICS |  | 695 17,429 | 75.2% 49.5% | <0.001 | 0.551 |
|  |  | 1 2 | CV100 | BETA BLOCKERS/RELATED |  | 474 5,482 | 51.3% 15.6% | <0.001 | 0.818 |
|  |  | 1 2 | CV700 | DIURETICS |  | 444 4,788 | 48.1% 13.6% | <0.001 | 0.804 |
|  |  | 1 2 | CV200 | CALCIUM CHANNEL BLOCKERS |  | 312 2,507 | 33.8% 7.1% | <0.001 | 0.700 |
|  |  | 1 2 | CV800 | ACE INHIBITORS |  | 326 3,194 | 35.3% 9.1% | <0.001 | 0.665 |
|  |  | 1 2 | CV805 | ANGIOTENSIN II INHIBITOR |  | 262 1,828 | 28.4% 5.2% | <0.001 | 0.652 |
|  |  | 1 2 | BL117 | PLATELET AGGREGATION INHIBITORS |  | 389 3,744 | 42.1% 10.6% | <0.001 | 0.765 |
|  |  | 1 2 | BL110 | ANTICOAGULANTS |  | 641 15,446 | 69.4% 43.9% | <0.001 | 0.533 |
| **Cohort 1 (N = 915) and cohort 2 (N = 915) characteristics after propensity score matching** | | | | | | | | | |
|  | **Demographics** | | | | | | | | |
|  |  | Cohort | |  | Mean ± SD | Patients | % of Cohort | P-Value | Std diff. |
|  |  | 1 2 | Age | Age | 68.1 +/- 10.1 68.7 +/- 10.7 | 915 915 | 100% 100% | 0.188 | 0.062 |
|  |  | 1 2 | 2106-3 | White |  | 612 636 | 66.9% 69.5% | 0.228 | 0.056 |
|  |  | 1 2 | F | Female |  | 895 893 | 97.8% 97.6% | 0.755 | 0.015 |
|  |  | 1 2 | 2054-5 | Black or African American |  | 169 161 | 18.5% 17.6% | 0.627 | 0.023 |
|  |  | 1 2 | 2028-9 | Asian |  | 18 12 | 2.0% 1.3% | 0.269 | 0.052 |
|  | **Diagnosis** | | | | | | | | |
|  |  | Cohort | |  | Mean ± SD | Patients | % of Cohort | P-Value | Std diff. |
|  |  | 1 2 | I10-I16 | Hypertensive diseases |  | 596 587 | 65.1% 64.2% | 0.660 | 0.021 |
|  |  | 1 2 | I20-I25 | Ischemic heart diseases |  | 164 141 | 17.9% 15.4% | 0.149 | 0.067 |
|  |  | 1 2 | I48 | Atrial fibrillation and flutter |  | 57 51 | 6.2% 5.6% | 0.552 | 0.028 |
|  |  | 1 2 | I50 | Heart failure |  | 71 61 | 7.8% 6.7% | 0.366 | 0.042 |
|  |  | 1 2 | E78 | Dyslipidemia |  | 688 695 | 75.2% 76.0% | 0.703 | 0.018 |
|  |  | 1 2 | E08-E13 | Diabetes mellitus |  | 313 315 | 34.2% 34.4% | 0.922 | 0.005 |
|  |  | 1 2 | E66 | Obesity |  | 307 292 | 33.6% 31.9% | 0.455 | 0.035 |
|  |  | 1 2 | N18 | Chronic kidney disease |  | 87 70 | 9.5% 7.7% | 0.156 | 0.066 |
|  |  | 1 2 | I63 | Cerebral infarction |  | 30 25 | 3.3% 2.7% | 0.494 | 0.032 |
|  | **Medication** | | | | | | | | |
|  |  | Cohort | |  | Mean ± SD | Patients | % of Cohort | P-Value | Std diff. |
|  |  | 1 2 | CV300 | ANTIARRHYTHMICS |  | 686 715 | 75.0% 78.1% | 0.110 | 0.075 |
|  |  | 1 2 | CV100 | BETA BLOCKERS/RELATED |  | 465 448 | 50.8% 49.0% | 0.427 | 0.037 |
|  |  | 1 2 | CV700 | DIURETICS |  | 435 441 | 47.5% 48.2% | 0.779 | 0.013 |
|  |  | 1 2 | CV200 | CALCIUM CHANNEL BLOCKERS |  | 303 287 | 33.1% 31.4% | 0.424 | 0.037 |
|  |  | 1 2 | CV800 | ACE INHIBITORS |  | 319 313 | 34.9% 34.2% | 0.768 | 0.014 |
|  |  | 1 2 | CV805 | ANGIOTENSIN II INHIBITOR |  | 254 228 | 27.8% 24.9% | 0.168 | 0.065 |
|  |  | 1 2 | BL117 | PLATELET AGGREGATION INHIBITORS |  | 380 369 | 41.5% 40.3% | 0.601 | 0.024 |
|  |  | 1 2 | BL110 | ANTICOAGULANTS |  | 632 647 | 69.1% 70.7% | 0.445 | 0.036 |

Supplementary Table 3. Baseline characteristics of breast cancer patients *lipophilic statin users* (cohort 1) and *statin non-users* (cohort 2) before and after propensity score matching.

| **Cohort 1 (N = 2,899) and cohort 2 (N = 37,553) characteristics before propensity score matching** | | | | | | | | | |
| --- | --- | --- | --- | --- | --- | --- | --- | --- | --- |
|  | **Demographics** | | | | | | | | |
|  |  | Cohort | |  | Mean ± SD | Patients | % of Cohort | P-Value | Std diff. |
|  |  | 1 2 | Age | Age | 69.0 +/- 10.4 59.6 +/- 12.8 | 2,748 35,216 | 100% 100% | <0.001 | 0.808 |
|  |  | 1 2 | 2106-3 | White |  | 1,842 19,748 | 67.0% 56.1% | <0.001 | 0.227 |
|  |  | 1 2 | F | Female |  | 2,687 34,766 | 97.8% 98.7% | <0.001 | 0.072 |
|  |  | 1 2 | 2054-5 | Black or African American |  | 463 4,065 | 16.8% 11.5% | <0.001 | 0.152 |
|  |  | 1 2 | 2028-9 | Asian |  | 89 991 | 3.2% 2.8% | 0.197 | 0.025 |
|  | **Diagnosis** | | | | | | | | |
|  |  | Cohort | |  | Mean ± SD | Patients | % of Cohort | P-Value | Std diff. |
|  |  | 1 2 | I10-I16 | Hypertensive diseases |  | 1,795 7,589 | 65.3% 21.5% | <0.001 | 0.984 |
|  |  | 1 2 | I20-I25 | Ischemic heart diseases |  | 450 1,001 | 16.4% 2.8% | <0.001 | 0.472 |
|  |  | 1 2 | I48 | Atrial fibrillation and flutter |  | 138 450 | 5.0% 1.3% | <0.001 | 0.216 |
|  |  | 1 2 | I50 | Heart failure |  | 183 548 | 6.7% 1.6% | <0.001 | 0.259 |
|  |  | 1 2 | E78 | Dyslipidemia |  | 1,970 4,818 | 71.7% 13.7% | <0.001 | 1.448 |
|  |  | 1 2 | E08-E13 | Diabetes mellitus |  | 946 2,459 | 34.4% 7.0% | <0.001 | 0.720 |
|  |  | 1 2 | E66 | Obesity |  | 842 4,203 | 30.6% 11.9% | <0.001 | 0.469 |
|  |  | 1 2 | N18 | Chronic kidney disease |  | 226 573 | 8.2% 1.6% | <0.001 | 0.308 |
|  |  | 1 2 | I63 | Cerebral infarction |  | 115 176 | 4.2% 0.5% | <0.001 | 0.245 |
|  | **Medication** | | | | | | | | |
|  |  | Cohort | |  | Mean ± SD | Patients | % of Cohort | P-Value | Std diff. |
|  |  | 1 2 | CV300 | ANTIARRHYTHMICS |  | 1,954 17,429 | 71.1% 49.5% | <0.001 | 0.453 |
|  |  | 1 2 | CV100 | BETA BLOCKERS/RELATED |  | 1,254 5,482 | 45.6% 15.6% | <0.001 | 0.690 |
|  |  | 1 2 | CV700 | DIURETICS |  | 1,316 4,788 | 47.9% 13.6% | <0.001 | 0.801 |
|  |  | 1 2 | CV200 | CALCIUM CHANNEL BLOCKERS |  | 816 2,507 | 29.7% 7.1% | <0.001 | 0.609 |
|  |  | 1 2 | CV800 | ACE INHIBITORS |  | 1,033 3,194 | 37.6% 9.1% | <0.001 | 0.716 |
|  |  | 1 2 | CV805 | ANGIOTENSIN II INHIBITOR |  | 612 1,828 | 22.3% 5.2% | <0.001 | 0.512 |
|  |  | 1 2 | BL117 | PLATELET AGGREGATION INHIBITORS |  | 1,243 3,744 | 45.2% 10.6% | <0.001 | 0.836 |
|  |  | 1 2 | BL110 | ANTICOAGULANTS |  | 1,694 15,446 | 61.6% 43.9% | <0.001 | 0.362 |
| **Cohort 1 (N = 2,676) and cohort 2 (N = 2,676) characteristics after propensity score matching** | | | | | | | | | |
|  | **Demographics** | | | | | | | | |
|  |  | Cohort | |  | Mean ± SD | Patients | % of Cohort | P-Value | Std diff. |
|  |  | 1 2 | Age | Age | 68.7 +/- 10.3 68.9 +/- 11.0 | 2,676 2,676 | 100% 100% | 0.597 | 0.014 |
|  |  | 1 2 | 2106-3 | White |  | 1,795 1,878 | 67.1% 70.2% | 0.014 | 0.067 |
|  |  | 1 2 | F | Female |  | 2,623 2,608 | 98.0% 97.5% | 0.168 | 0.038 |
|  |  | 1 2 | 2054-5 | Black or African American |  | 443 431 | 16.6% 16.1% | 0.657 | 0.012 |
|  |  | 1 2 | 2028-9 | Asian |  | 87 86 | 3.3% 3.2% | 0.938 | 0.002 |
|  | **Diagnosis** | | | | | | | | |
|  |  | Cohort | |  | Mean ± SD | Patients | % of Cohort | P-Value | Std diff. |
|  |  | 1 2 | I10-I16 | Hypertensive diseases |  | 1,724 1,719 | 64.4% 64.2% | 0.887 | 0.004 |
|  |  | 1 2 | I20-I25 | Ischemic heart diseases |  | 408 370 | 15.2% 13.8% | 0.141 | 0.040 |
|  |  | 1 2 | I48 | Atrial fibrillation and flutter |  | 133 120 | 5.0% 4.5% | 0.402 | 0.023 |
|  |  | 1 2 | I50 | Heart failure |  | 173 151 | 6.5% 5.6% | 0.207 | 0.034 |
|  |  | 1 2 | E78 | Dyslipidemia |  | 1,898 1,955 | 70.9% 73.1% | 0.083 | 0.047 |
|  |  | 1 2 | E08-E13 | Diabetes mellitus |  | 891 842 | 33.3% 31.5% | 0.152 | 0.039 |
|  |  | 1 2 | E66 | Obesity |  | 810 781 | 30.3% 29.2% | 0.386 | 0.024 |
|  |  | 1 2 | N18 | Chronic kidney disease |  | 214 184 | 8.0% 6.9% | 0.118 | 0.043 |
|  |  | 1 2 | I63 | Cerebral infarction |  | 105 82 | 3.9% 3.1% | 0.087 | 0.047 |
|  | **Medication** | | | | | | | | |
|  |  | Cohort | |  | Mean ± SD | Patients | % of Cohort | P-Value | Std diff. |
|  |  | 1 2 | CV300 | ANTIARRHYTHMICS |  | 1,898 1,976 | 70.9% 73.8% | 0.017 | 0.065 |
|  |  | 1 2 | CV100 | BETA BLOCKERS/RELATED |  | 1,195 1,181 | 44.7% 44.1% | 0.700 | 0.011 |
|  |  | 1 2 | CV700 | DIURETICS |  | 1,253 1,206 | 46.8% 45.1% | 0.197 | 0.035 |
|  |  | 1 2 | CV200 | CALCIUM CHANNEL BLOCKERS |  | 766 739 | 28.6% 27.6% | 0.412 | 0.022 |
|  |  | 1 2 | CV800 | ACE INHIBITORS |  | 975 928 | 36.4% 34.7% | 0.180 | 0.037 |
|  |  | 1 2 | CV805 | ANGIOTENSIN II INHIBITOR |  | 573 560 | 21.4% 20.9% | 0.664 | 0.012 |
|  |  | 1 2 | BL117 | PLATELET AGGREGATION INHIBITORS |  | 1,172 1,091 | 43.8% 40.8% | 0.025 | 0.061 |
|  |  | 1 2 | BL110 | ANTICOAGULANTS |  | 1,651 1,690 | 61.7% 63.2% | 0.271 | 0.030 |
